# External validity of Adult Sepsis Event’s simplified eSOFA criteria: a retrospective analysis of patients with confirmed infection in China

**DOI:** 10.1186/s13613-020-0629-1

**Published:** 2020-02-04

**Authors:** Run Dong, Hongcheng Tian, Jianfang Zhou, Li Weng, Xiaoyun Hu, Jinmin Peng, Chunyao Wang, Wei Jiang, Xueping Du, Xiuming Xi, Youzhong An, Meili Duan, Bin Du, for the China Critical Care Clinical Trials Group (CCCCTG)

**Affiliations:** 1 Medical ICU, Peking Union Medical College Hospital, Peking Union Medical College & Chinese Academy of Medical Sciences, 1 Shuai Fu Yuan, Beijing, 100730 People’s Republic of China; 2 Department of Critical Care Medicine, China Rehabilitation Research Center, Capital Medical University, 10 Jiaomen Beilu, Fengtai District, Beijing, 100068 People’s Republic of China; 3 Department of Critical Care Medicine, Beijing Tian Tan Hospital, Capital Medical University, 6 Tiantan Xili, Beijing, 100050 People’s Republic of China; 4 Department of General Internal Medicine, Fuxing Hospital, Capital Medical University, A20 Fu Xing Men Wai Street, Beijing, 100038 People’s Republic of China; 5 Department of Critical Care Medicine, Fuxing Hospital, Capital Medical University, A20 Fu Xing Men Wai Street, Beijing, 100038 People’s Republic of China; 6 Department of Critical Care Medicine, Peking University People’s Hospital, 11 Xi Zhi Men South Street, Beijing, 100044 People’s Republic of China; 7 Department of Critical Care Medicine, Beijing Friendship Hospital, Capital Medical University, 95 Yong’an Road, Beijing, 100050 People’s Republic of China

**Keywords:** Sepsis, Surveillance, Adult Sepsis Event, Sequential Organ Failure Assessment Score

## Abstract

**Background:**

The US Centers for Disease Control and Prevention (CDC) recently released simplified eSOFA organ dysfunction criteria of Adult Sepsis Event for sepsis surveillance in the US. Our study aimed to compare the prevalence, characteristics, and outcomes of sepsis patients identified by eSOFA criteria versus Sequential Organ Failure Assessment (SOFA) Score (Sepsis-3) and assess the external validity of eSOFA criteria in China.

**Methods:**

We conducted a retrospective cohort study of adult residents of Yuetan Subdistrict, Beijing, China, who were hospitalized from July 1, 2012 to June 30, 2014. Among patients with infection, sepsis was identified if there was a concurrent rise in SOFA score by 2 or more points (Sepsis-3) or the presence of 1 or more eSOFA criteria: vasopressor initiation, mechanical ventilation initiation, doubling in creatinine, doubling in bilirubin to 2.0 mg/dL or above, 50% or greater decrease in platelet count to less than 100 cells/μL, or lactate equal to or above 2.0 mmol/L. Areas under the receiver operating characteristic curves (AUROCs) for in-hospital mortality were compared between sepsis patients detected by the two criteria, adjusting for baseline characteristics.

**Results:**

Of 1716 hospitalized patients with infection, 935 (54.5%) met Sepsis-3 criteria, 573 (33.4%) met eSOFA criteria, while 475 (27.7%) met both criteria. Demographic and clinical characteristics of sepsis patients meeting Sepsis-3 or eSOFA criteria were similar. In-hospital mortality was higher with eSOFA criteria versus Sepsis-3 (46.6% vs. 32.0%, *p* < 0.001). eSOFA criteria had high PPV (82.9%), but low sensitivity (50.8%) for the diagnosis of Sepsis-3. Patients meeting both criteria had the highest in-hospital mortality rate (52.8%, all *p* < 0.001), while patients who only met eSOFA criteria had higher mortality rate than those meeting Sepsis-3 alone (16.3% vs. 10.4%, *p* = 0.097). The predicted probability for in-hospital mortality was higher with eSOFA criteria versus Sepsis-3 (AUROC 0.830 vs. 0.795, *p* = 0.001) adjusting for baseline characteristics.

**Conclusions:**

The CDC Adult Sepsis Event’s eSOFA criteria identify a smaller, more severely ill cohort of sepsis patients with similar demographic and clinical characteristics as the more complex Sepsis-3 SOFA score. These results suggest similar performance of eSOFA criteria across diverse populations, with low sensitivity and high specificity for the diagnosis of Sepsis-3.

## Introduction

Sepsis remains the leading cause of death in critically ill patients, with over 1.7 million adult sepsis cases annually in the US which contribute to 270,000 deaths [1]. In China, standardized sepsis-related mortality rate was 66.7 deaths per 100,000 population, producing a national estimate of 1,025,997 sepsis-related deaths in 2015 [2].

The Third International Consensus Definitions Task Force defined sepsis as a “life-threatening organ dysfunction due to a dysregulated host response to infection”, and recommended use of an acute increase in Sequential Organ Failure Assessment (SOFA) Score by 2 or more points as the working definition (Sepsis-3) to identify the presence of organ dysfunction [3]. However, alternative sepsis criteria serve different purposes, including clinical care, research, surveillance, and quality improvement and audit [4]. Sepsis-3 based on SOFA score was chosen for clinical care due to their superior content and criterion validity as well as good timeliness [5], but did not perfectly suit the purpose of surveillance since many components are not routinely or consistently recorded [6]. Moreover, it is particularly difficult to calculate SOFA score from the electronic health records (EHRs) which is not dedicated for this purpose.

In 2018, the US Centers for Disease Control and Prevention (CDC) proposed the criteria of Adult Sepsis Event, based on organ dysfunction criteria (eSOFA) analogous to SOFA score (Sepsis-3) [7]. It was developed for retrospective surveillance using objective data that can be directly obtained from EHRs. Rhee et al. have compared sepsis patients detected by eSOFA criteria with those identified by Sepsis-3 and validated the use of eSOFA for sepsis surveillance in the US [1, 6]. It is important to assess the external validity of eSOFA criteria in other countries. However, the predictive validity only represented one, although the most commonly studied, of the six domains of usefulness for sepsis criteria [4, 8]. As a practical, simplified adaptation of Sepsis-3 that was newly proposed for consistent, automated sepsis surveillance, it is also crucial to understand the prevalence, clinical characteristics and outcomes of sepsis patients who were missed by eSOFA criteria (i.e., false negatives) and those misdiagnosed as sepsis (i.e., false positives).

In this retrospective study, we compared the prevalence, characteristics, and outcomes of sepsis patients detected using eSOFA (CDC Adult Sepsis Event) versus SOFA score (Sepsis-3) in a database of patients with Sepsis-1 from a subdistrict of Beijing. In addition, we also investigated characteristics and outcomes of false negatives and false positives based on eSOFA criteria. We hypothesized that CDC Adult Sepsis Event’s simplified eSOFA criteria could perform comparably as Sepsis-3 in terms of detecting sepsis patients and predicting mortality, which supports its use as a practical tool for sepsis surveillance.

## Methods

### Study design, data source, and definitions

This was a retrospective analysis of a database of 1716 patients fulfilling Sepsis-1 criteria, with data source and definitions of the study described in a previous study [9]. We conducted a retrospective cohort study of all adult residents (≥ 18 years old) of Yuetan Subdistrict, Beijing, China, who were hospitalized from July 1, 2012 to June 30, 2014. Medical records of these patients were identified from the hospital discharge database of Beijing Public Health Information System and manually reviewed independently by any two of three investigators each with more than 5 years of ICU working experience. Any disagreement was resolved by discussion. Final decision was made by the steering committee (XM, YA, and BD) if consensus could not be reached.

Patients with any of the following definitions were identified as infected. Community-acquired infection was identified based on clinical, imaging, and microbiologic parameters, whereas nosocomial infection was diagnosed according to the set of standardized definitions of the CDC [10]. Microbiologically documented infection was confirmed by positive cultures of blood or body fluid from a site of suspected infection, and patients with the presence of gross purulence or an abscess (anatomical and/or by imaging and/or histologic evidence), but without a microbiologic documentation, were considered to have clinically documented infection.

The US CDC released the Adult Sepsis Event (eSOFA) as simpler criteria that include the same organ systems as SOFA score except replacing Glasgow Coma Score (GCS) by lactate greater than or equal to 2.0 mmol/L [7]. The eSOFA criteria include the following organ dysfunctions: (1) vasopressor initiation; (2) initiation of mechanical ventilation; (3) doubling of serum creatinine or decrease by 50% of estimated glomerular filtration rate (eGFR) relative to baseline, excluding patients with end-stage renal disease; (4) total bilirubin ≥ 2.0 mg/dL and increase by 100% from baseline; (5) platelet count < 100 cells/μL and ≥ 50% decline from baseline (baseline must be ≥ 100 cells/μL); (6) serum lactate ≥ 2.0 mmol/L. For patients with infection, we calculated maximum eSOFA and SOFA score based on retrieved clinical data until 72 h after hospital admission (for those who were admitted due to infection) or onset of infection (for those who developed infection during hospitalization). We identified a hospital admission as having sepsis if there was infection and concurrent organ dysfunction defined by either the presence of 1 or more eSOFA criteria [1, 6, 7] or a rise in SOFA score by 2 or more points (Sepsis-3) [3].

Missing data imputation for eSOFA or SOFA score were performed based on relevant information in the medical records, surrogate markers, or data obtained before and after data collection date. If none of these were available, we recorded the missing variable as zero for the corresponding category of organ dysfunction in the final analysis. For example, we considered free text such as no jaundice in the medical records as surrogates for normal serum bilirubin level, or consciousness as indication of normal mentation. Moreover, in cases without arterial blood gas, we substituted SpO_2_/FiO_2_ ratio for PaO_2_/FiO_2_ ratio [11]. However, we did not perform missing data imputation for lactate due to the lack of reliable surrogate markers.

### Statistical analysis

We examined the prevalence, characteristics, and outcomes of sepsis patients defined by either eSOFA or Sepsis-3 criteria. Crude mortality rates were compared using two-sample *z*-test. The sensitivity, specificity, and positive predictive value (PPV) of eSOFA criteria were calculated comparing to Sepsis-3 criteria. The agreement between sepsis patients identified by SOFA and eSOFA criteria was examined using Cronbach’s alpha [3, 12].

The predictive values of Sepsis-3 and eSOFA criteria for in-hospital mortality were compared by the area under the receiver operation characteristics (AUROC) curves with DeLong method, with and without adjustment for covariates in multivariate logistic regression analysis. In addition, in-hospital mortality rates of patient groups classified by Sepsis-3 and eSOFA criteria (i.e., Sepsis-3−/eSOFA−, Sepsis-3+/eSOFA−, Sepsis-3−/eSOFA+ and Sepsis-3+/eSOFA+) were also compared by multivariate logistic regression analysis. Potential risk factors added into the model included demographics (age and gender), body mass index (BMI), Charlson Comorbidity Index, and characteristics of infection. Age was categorized into three categories (18–64, 65–84, and ≥ 85 years), because the assumption of linearity would be violated if age was included in the model as a continuous variable [13, 14]. Akaike information criterion (AIC) was used to measure the relative quality of the models.

Continuous variables were presented as median and interquartile range (IQR). Categorical variables were presented as a percentage of the group from which they were derived, and compared by the use of Chi-square test or Fisher’s exact test. All comparisons were unpaired and all tests of significance were two-tailed. Analyses were conducted using SPSS version 22. A *p* value < 0.05 was considered as statistically significant.

### Ethical approval

This study was approved by the ethics committee of Peking Union Medical College Hospital and informed consent was waived. This study was registered at ClinicalTrials.gov, with registration number NCT02285257.

## Results

### Prevalence, characteristics, and in-hospital mortality of sepsis patients defined by eSOFA or Sepsis-3 criteria

During the study period, 22,552 Yuetan residents were admitted into any of the 111 hospitals within the Beijing Public Health Information System, of whom the medical records of 21,191 admissions were manually reviewed. We were unable to review the medical records of the other 1361 admissions either because of missing records (*n* = 277) or refusal by the hospitals (*n* = 1084). A total of 1716 patients meeting Sepsis-1 criteria were identified from 3449 patients with infection, and were included in the final analysis.

Among the 1716 infected patients, 935 (54.5%) met Sepsis-3 criteria, 573 (33.4%) met CDC Adult Sepsis Event eSOFA criteria, while 475 (27.7%) met both criteria. The agreement between eSOFA and Sepsis-3 criteria was moderate with Cronbach’s alpha 0.56. Frequency of missing variables and missing data imputation for SOFA and eSOFA criteria is shown in Additional file 1: Table S1.

Demographics, comorbidities, and clinical characteristics of sepsis patients meeting Sepsis-3 or eSOFA criteria were generally similar (Table 1). Compared with those who did not meet Sepsis-3 criteria, patients defined by Sepsis-3 criteria were older, more likely to be male, and prone to be complicated with chronic heart, pulmonary, or renal diseases. Similar differences were also found comparing patients meeting eSOFA criteria or not (Table 1). Pneumonia and intra-abdominal infections were the most common sites of infection for both sets of septic patients. Respiratory and coagulation dysfunction were the most common organ dysfunctions in sepsis patients defined by Sepsis-3, whereas respiratory dysfunction and elevated lactate were the most common organ dysfunctions in patients meeting eSOFA criteria (Additional file 1: Table S2).

**Table 1:** Characteristics of infected patients with or without sepsis defined by Sepsis-3 or eSOFA criteria

Characteristic	eSOFA (−) *n* = 1143	eSOFA (+) *n* = 573	Sepsis-3 (−) *n* = 781	Sepsis-3 (+) *n* = 935
Median age (IQR)	79 (62–84)	82 (75–87)**	78 (57–84)	81 (74–86)^‡^
Gender, *n* (%)				
Male	642 (56.2)	346 (60.4)	414 (53.0)	574 (61.4)^‡^
BMI, kg/m^2^, median (IQR)	23 (20–26)	23 (20–26)	24 (21–26)	23 (20–26)^†^
Bedridden, *n* (%)	580 (50.7)	310 (54.1)**	388 (49.7)	502 (53.7)^‡^
Type of hospital admission, *n* (%)				
Medical	1047 (91.6)	506 (88.3)*	691 (88.5)	862 (92.2)^‡^
Elective surgery	85 (7.4)	43 (7.5)	75 (9.6)	53 (5.7)^‡^
Emergency surgery	11 (1.0)	24 (4.2)**	15 (1.9)	20 (2.1)
McCabe and Jackson classification, *n* (%)				
Nonfatal	814 (71.2)	394 (68.8)	524 (67.1)	684 (73.2)^‡^
Ultimately fatal	169 (14.8)	120 (20.9)**	112 (14.3)	177 (18.9)^†^
Rapidly fatal	21 (1.8)	24 (4.2)**	22 (2.8)	23 (2.5)
Charlson Comorbidity Index, median (IQR)	1 (1–3)	2 (1–3)**	1 (0–3)	2 (1–3)^‡^
Comorbidities (Charlson), *n* (%)				
Cancer^a^	174 (15.2)	131 (22.9)**	133 (17.0)	172 (18.4)
Congestive heart failure	12 (1.0)	28 (4.9)**	9 (1.2)	31 (3.3)^‡^
Chronic pulmonary disease	237 (20.7)	148 (25.8)*	150 (19.2)	235 (25.1)^‡^
Diabetes	328 (28.7)	126 (22.0)**	206 (26.4)	248 (26.5)
Liver disease	15 (1.3)	17 (3.0)*	13 (1.7)	19 (2.0)
Renal disease	46 (4.0)	31 (5.4)	10 (1.3)	67 (7.2)^‡^
Chronic organ dysfunction (APACHE II), *n* (%)				
Cardiovascular	11 (1.0)	26 (4.5)**	8 (1.0)	29 (3.1)‡
Respiratory	37 (3.2)	70 (12.2)**	42 (5.4)	65 (7.0)
Liver	15 (1.3)	17 (3.0)*	13 (1.7)	19 (2.0)
Renal	34 (3.0)	21 (3.7)	10 (1.3)	45 (4.8)^‡^
Immunosuppression	94 (8.2)	57 (9.9)	62 (7.9)	89 (9.5)
Site of infection, *n* (%)				
Pneumonia	660 (57.7)	374 (65.3)**	432 (55.3)	602 (64.4)^‡^
Urogenital tract infection	90 (7.9)	11 (1.9)**	68 (8.7)	33 (3.5)^‡^
Intra-abdominal infection	105 (9.2)	72 (12.6)*	81 (10.4)	96 (10.3)
Skin/soft tissue infection	25 (2.2)	3 (0.5)*	24 (3.1)	4 (0.4)^‡^
Septicemia/bacteremia	18 (1.6)	13 (2.3)	18 (2.3)	13 (1.4)
Two or more infections	61 (5.3)	66 (11.5)**	55 (7.0)	72 (7.7)
Outcomes				
Median hospital LOS (IQR)	17 (9–30)	23 (11–40)*	17 (9–29)	20 (11–38)^†^
Required ICU admission, *n* (%)	33 (2.9)	218 (38.1)**	16 (2.0)	235 (25.1)^‡^
Death, *n* (%)	86 (7.5)	267 (46.6)**	54 (6.9)	299 (32.0)^‡^

*IQR* interquartile range, *BMI* body mass index, *LOS* length of stay

*p < 0.05, **p < 0.01, compared with eSOFA (−); ^†^*p* < 0.05, ^‡^*p* < 0.01, compared with Sepsis-3 (−)

^a^Cancer includes solid tumor with or without metastases, leukemia, and lymphoma

Crude in-hospital mortality rates were higher in patients meeting eSOFA criteria than those meeting Sepsis-3 criteria (46.6% vs. 32.0%; *p* < 0.001). The predictive value of eSOFA criteria for in-hospital mortality was significantly higher than that of Sepsis-3 criteria, with (AUROC 0.830 [95% CI 0.812–0.848] vs. 0.795 [0.775–0.814]; *p* = 0.001) and without (0.762 [0.742–0.782] vs. 0.690 [0.668–0.712]; *p* < 0.001) adjustment for demographics, BMI, Charlson Comorbidity Index and site of infection.

### Prevalence, characteristics, and in-hospital mortality of Sepsis-3+/eSOFA−, Sepsis-3−/eSOFA+, and Sepsis-3+/eSOFA+ patients

Infected patients who met Sepsis-3 but not eSOFA criteria (*n* = 460) were less likely to have comorbidities such as cancer, chronic respiratory dysfunction, and chronic heart disease compared with patients who met both criteria (Table 2). In terms of organ dysfunctions flagged by SOFA score, Sepsis-3+/eSOFA− patients tended to have mild hypoxia that did not require mechanical ventilation, mild coagulation dysfunction, or elevated creatinine that did not reach twice of baseline creatinine (Additional file 1: Table S2).

**Table 2:** Characteristics of sepsis patients categorized by overlap of Sepsis-3 and eSOFA criteria (Adult Sepsis Event)

Characteristic	Sepsis-3 (−) eSOFA (−) *n* = 683	Sepsis-3 (+) eSOFA (−) *n* = 460	Sepsis-3 (−) eSOFA (+) *n* = 98	Sepsis-3 (+) eSOFA (+) *n* = 475
Median age (IQR)	77 (56–84)	81 (73–85)**	81 (73–86)**^‡^	82 (75–87)**
Gender, *n* (%)				
Male	359 (52.6)	283 (61.5)**	43 (43.9)	291 (61.3)**
BMI, kg/m^2^, median (IQR)	24 (21–26)	23 (20–26)*	24 (20–25)	23 (20–26)*
Bedridden, *n* (%)	336 (49.2)	242 (52.6)**	52 (53.1)	260 (54.7)**
Type of hospital admission, *n* (%)				
Medical	610 (89.3)	437 (95.0)**^‡^	81 (82.7)	425 (89.5)
Elective surgery	66 (9.7)	19 (4.1)**^†^	9 (9.2)	34 (7.2)
Emergency surgery	7 (1.0)	4 (0.9)^‡^	8 (8.2)**^†^	16 (3.4)**
McCabe and Jackson classification, *n* (%)				
Nonfatal	464 (67.9)	350 (76.1)**^†^	60 (61.2)	334 (70.3)
Ultimately fatal	90 (13.2)	79 (17.2)	22 (22.4)*	98 (20.6)**
Rapidly fatal	14 (2.0)	7 (1.5)	8 (8.2)**^†^	16 (3.4)
Charlson Comorbidity Index, median (IQR)	1 (0–3)	2 (1–3)**^‡^	2 (1–3)**	2 (1–3)**
Comorbidities (Charlson), *n* (%)				
Cancer^a^	103 (15.1)	71 (15.4)^†^	30 (30.6)**^†^	101 (21.3)**
Congestive heart failure	5 (0.7)	7 (1.5)^‡^	4 (4.1)**	24 (5.1)**
Chronic pulmonary disease	121 (17.7)	116 (25.2)**	29 (29.6)**	119 (25.1)**
Diabetes	181 (26.5)	147 (32.0)*^‡^	25 (25.5)	101 (21.3)*
Liver disease	9 (1.3)	6 (1.3)	4 (4.1)*	13 (2.7)
Renal disease	7 (1.0)	39 (8.5)**	3 (3.1)	28 (5.9)**
Chronic organ dysfunction (APACHE II), *n* (%)				
Cardiovascular	4 (0.6)	7 (1.5)^‡^	4 (4.1)**	22 (4.6)**
Respiratory	16 (2.3)	21 (4.6)*^‡^	26 (26.5)**^‡^	44 (9.3)**
Liver	9 (1.3)	6 (1.3)	4 (4.1)*	13 (2.7)
Renal	6 (0.9)	28 (6.1)**	4 (4.1)**	17 (3.6)**
Immunosuppression	56 (8.2)	38 (8.3)	6 (6.1)	51 (10.7)
Site of infection, *n* (%)				
Pneumonia	369 (54.0)	291 (63.3)**	63 (64.3)	311 (65.5)**
Urogenital tract infection	65 (9.5)	25 (5.4)*^‡^	3 (3.1)*	8 (1.7)**
Intra-abdominal infection	72 (10.5)	33 (7.2)^‡^	9 (9.2)	63 (13.3)
Skin/soft tissue infection	22 (3.2)	3 (0.7)**	2 (2.0)†	1 (0.2)**
Septicemia/bacteremia	15 (2.2)	3 (0.7)*	3 (3.1)	10 (2.1)
Two or more infections	41 (6.0)	20 (4.3)^‡^	14 (14.3)**	52 (10.9)**
Outcomes				
Median hospital LOS (IQR)	17 (9–28)	17 (10–33)^‡^	23 (11–38)*	23 (11–41)**
Required ICU admission, *n* (%)	7 (1.0)	26 (5.7)**^‡^	9 (9.2)**^‡^	207 (43.6)**
Death, n (%)	38 (5.6)	48 (10.4)**^‡^	16 (16.3)**^‡^	251 (52.8)**

*IQR* interquartile range, *BMI* body mass index, *LOS* length of stay

*p < 0.05, **p < 0.01, compared with eSOFA(−)Sepsis(−); ^†^*p* < 0.05, ^‡^*p* < 0.01, compared with eSOFA(+)Sepsis(+)

^a^Cancer includes solid tumor with or without metastases, leukemia, and lymphoma

Meanwhile, infected patients who met eSOFA but not Sepsis-3 criteria (*n* = 98) were more likely to be complicated by cancer or chronic respiratory dysfunction compared with sepsis patients meeting both criteria (Table 2). Among the 98 eSOFA+/Sepsis-3− patients, 30 (30.6%) had elevated lactate alone without other organ dysfunctions (Additional file 1: Table S2). Mechanical ventilation was initiated for 24 (24.5%) patients, whose PaO_2_/FiO_2_ were not measured (*n* = 9) or did not fall under 300 (i.e., respiratory SOFA score ≤ 1) (*n* = 15). Detailed description of eSOFA/SOFA organ dysfunctions of eSOFA+/Sepsis-3− patients is presented in Additional file 1: eResults.

Sepsis patients who met both Sepsis-3 and eSOFA criteria tended to have elevated lactate and high rates of cardiovascular and respiratory dysfunction (Additional file 1: Figure S1). Patients in Sepsis-3−/eSOFA− group had the lowest mortality rate (5.6%, all *p* < 0.001). In addition, patients in Sepsis-3−/eSOFA+ group had a higher mortality rate than those in Sepsis-3+/eSOFA− group (16.3% vs. 10.4%; *p* = 0.097), with adjusted odds ratio (OR) of 2.45 (95% CI 1.27–4.73) and 2.09 (95% CI 1.35–3.25), respectively. In comparison, patients in Sepsis-3+/eSOFA+ group had the highest mortality rate (52.8%, all *p* < 0.001), with adjusted OR of 17.20 (95% CI 11.52–25.67) (Additional file 1: Table S3).

The sensitivity of Adult Sepsis Event eSOFA Criteria for Sepsis-3 diagnosis was 50.8%, the specificity was 87.5%, and PPV was 82.9%.

## Discussion

In this population-based cohort in China, we found that CDC Adult Sepsis Event eSOFA criteria identified a smaller group of sepsis patients with higher mortality compared to Sepsis-3 criteria. These findings were consistent with previous study in US hospitals that reported lower prevalence (4.4% vs. 6.1%) and higher mortality (17.1% vs. 14.4%) of sepsis patients defined by eSOFA versus Sepsis-3 criteria [6].

In our study, eSOFA criteria had high PPV (82.9%). Only 98 patients (false positive rate 12.5%) were misdiagnosed as sepsis by eSOFA criteria, with hyperlactatemia without evidence of concurrent organ dysfunctions being the major cause. Moreover, the mortality rate of these patients was significantly higher than those fulfilling neither Sepsis-3 nor eSOFA criteria (16.3% vs. 5.6%) [15–17]. This indicates that SOFA score alone is unable to detect all infected patients with high risk of mortality [18], and further studies are needed to assess whether additional screening parameters such as lactate could be helpful. In comparison, eSOFA criteria identified more severely ill patients, possibly by including invasive procedures (such as vasoactive agents or invasive mechanical ventilation) in the criteria, although they might not be the same patients diagnosed as septic at the bedside. Meanwhile, our study presented a severely ill cohort of infected patients with older age, more pneumonia (60.3%), higher rates of mechanical ventilation (13.6%) and vasopressor use (7.9%), which might explain the high PPV.

The sensitivity of eSOFA criteria (50.8%) was considerably reduced by patients with mild hypoxia that did not reach the eSOFA point of mechanical ventilation, those with mild thrombocytopenia that did not reach a decrease of platelets by 50% or more from baseline, as well as those with elevated creatinine that was lower than the doubling of baseline. As a result, eSOFA criteria might miss the diagnosis of septic patients who are less severely ill but with significant risk of in-hospital mortality (adjusted OR 2.09, 95%CI 1.35–3.25), questioning the potential use of eSOFA alone as a surveillance tool for sepsis [6].

Our study has some strengths. First, no study has evaluated the external validity of eSOFA criteria or investigated the characteristics and outcomes of infected patients missed and misdiagnosed by eSOFA criteria. Moreover, we manually reviewed the medical records of all patients to obtain relevant data and made diagnosis of infection and sepsis using standardized definitions, whereas previous studies used EHR-based proxies as culture orders and antibiotic administrations for infection [3, 6].

Our study has several limitations. First, this study is a secondary analysis of a database that was not originally designed for the study purpose. Among the 3449 patients with infection in the original cohort, we only collected data from 1716 patients who met Sepsis-1 criteria. This might introduce selection bias to the current study. However, Luo et al. reported that, among 38 infected patients who did not fulfill Sepsis-1 criteria, only 5 were diagnosed as Sepsis-3 [18]. Therefore, it was unlikely that addition of these patients in our analysis would change the major results. Second, the mortality rates of septic patients were significantly higher than those in Rhee et al.’s study. Older age (median age 82 for eSOFA+ patients, and 81 for Sepsis-3+ patients in our study) and higher rate of pneumonia might cause the difference since they were proved as independent risk factors for mortality of sepsis patients [4, 5, 19, 20]. Meanwhile, mortality rates of septic patients vary in previous studies by factors including age, sex, comorbidities, and acuity of illness [2, 19–22]. Third, our cohort represented a patient population significantly different from the original patient population from which eSOFA criteria were developed, as suggested by older age, more pneumonia, higher rate of mechanical ventilation, and higher mortality. Although our results might not be generalized to other patient populations, it clearly demonstrated the same advantages and disadvantages of eSOFA criteria, i.e., external validity, in different settings and patients [6]. Fourth, some data necessary for the calculation of SOFA and/or eSOFA score were missing, particularly with high proportion of missing data from lactate values. However, the frequency of missing data in our cohort was comparable to that in previous studies [3, 6]. As a matter of fact, this might be regarded as limitations of the scoring systems, rather than limitations of the current and previous studies, because both eSOFA and SOFA scoring systems contained variables such as lactate and, possibly, bilirubin that were not routinely monitored in general wards. Last, due to the lack of gold standard for diagnosis of sepsis [4, 5, 23, 24], concordance between eSOFA and Sepsis-3 criteria, rather than sensitivity and specificity, should be reported. However, in order to highlight the limitations of applying eSOFA criteria as a surrogate of SOFA score, as well as to compare our results with those in previous study [6], we still calculated sensitivity, specificity, and PPV of eSOFA criteria compared to Sepsis-3.

## Conclusion

In conclusion, we found that, similar to the study in the US hospitals, the CDC Adult Sepsis Event’s simplified eSOFA organ dysfunction criteria identify a smaller cohort of sepsis patients with similar demographic and clinical characteristics as those identified using Sepsis-3 SOFA score, but with higher risk of death. These results suggest similar performance of eSOFA criteria across diverse populations. However, the poor prognosis of patients with Sepsis-3 who are missed by eSOFA criteria might limit the use of eSOFA criteria as a surveillance tool for sepsis.

## Supplementary information

**Additional file 1: Table S1**. Imputation of missing data for the calculation of SOFA or eSOFA score. **eResults.** Detailed Description of eSOFA/SOFA score of the 98 eSOFA+/Sepsis-3− patients**. Table S2.** Frequency of SOFA/eSOFA organ dysfunctions in patients meeting Sepsis-3 or eSOFA criteria. **Figure S1.** Frequency of SOFA or eSOFA organ dysfunctions in Sepsis-3+/eSOFA+ sepsis patients. **Table S3.** Risk models of in-hospital mortality.

## Data Availability

The datasets used and/or analyzed during the current study are available from the corresponding author on reasonable request.

## Abbreviations

SOFA: Sequential Organ Failure Assessment
CDC: Centers for Disease Control and Prevention
EHRs: Electronic health records
GCS: Glasgow Coma Score
eGFR: Estimated glomerular filtration rate
PPV: Positive predictive value
AUROC: Area under the receiver operation characteristics
IQR: Interquartile range
BMI: Body mass index
AIC: Akaike information criterion
OR: Odds ratio
