# Supplementary material for: External validity of Adult Sepsis Event’s simplified eSOFA criteria: a retrospective analysis of patients with confirmed infection in China
Source: Ann Intensive Care. 2020 Feb 4;10:14. doi: 10.1186/s13613-020-0629-1 (PMC7000563; doi:10.1186/s13613-020-0629-1)
Supplement: Supplementary file 1 — Additional file 1: Table S1. Imputation of missing data for the calculation of SOFA or eSOFA score. eResults. Detailed Description of eSOFA/SOFA score of the 98 eSOFA+/Sepsis-3− patients. Table S2. Frequency of SOFA/eSOFA organ dysfunctions in patients meeting Sepsis-3 or eSOFA criteria. Figure S1. Frequency of SOFA or eSOFA organ dysfunctions in Sepsis-3+/eSOFA+ sepsis patients. Table S3. Risk models of in-hospital mortality. [file 13613_2020_629_MOESM1_ESM.docx]

**Additional information**

**External Validity of Adult Sepsis Event’s Simplified eSOFA Criteria: A Retrospective Analysis of Patients with Confirmed Infection in China**

**Table S1** Imputation of missing data for the calculation of SOFA or eSOFA score

| Variable | Original data missing | Data imputation* | Data missing in final analysis** |
| --- | --- | --- | --- |
| Lactate (eSOFA) | 55.4% (951/1716) | 0 | 55.4% (951/1716) |
| PaO_2_/FiO_2_ ratio (SOFA) | 43.6% (749/1716) | 20.7% (356/1716) | 22.9% (393/1716) |
| Glasgow Coma Scale (SOFA) | 12.3% (211/1716) | 8.4% (145/1716) | 3.8% (66/1716) |
| Hepatic dysfunction (SOFA and eSOFA) | 5.5% (94/1716) | 2.4% (42/1716) | 3.0% (52/1716) |
| Coagulation dysfunction (SOFA and eSOFA) | 5.1% (87/1716) | 0.7% (12/1716) | 4.4% (75/1716) |
| Renal dysfunction (SOFA and eSOFA) | 1.9% (33/1716) | 0.2% (3/1716) | 1.7% (30/1716) |
| Cardiovascular dysfunction (SOFA) | 0.3% (5/1716) | 0.1% (2/1716) | 0.2% (3/1716) |

*data imputation based on relevant information, such as medical records, surrogate markers, and laboratory tests before and after data collection date

** recorded as zero for the corresponding category of organ dysfunction

Missing data imputation for eSOFA or SOFA score were performed based on relevant information in the medical records, surrogate markers, or data obtained before and after data collection date. If none of these were available, we recorded the missing variable as zero for the corresponding category of organ dysfunction in the final analysis. For example, we considered free text such as no jaundice in the medical records as surrogates for normal serum bilirubin level, or consciousness as indication of normal mentation. Moreover, in cases without arterial blood gas, we substituted SpO_2_/FiO_2_ ratio for PaO_2_/FiO_2_ ratio. However, we did not perform missing data imputation for lactate due to the lack of reliable surrogate markers.

**eResults – Detailed Description of eSOFA/SOFA score of the 98 eSOFA+/Sepsis-3- patients**

1. 30 had elevated lactate alone without evidence of other organ dysfunctions.
2. 24 received mechanical ventilation, whose PaO_2_/FiO_2_ were not measured (n=9) or did not fall under 300 (respiratory SOFA score ≤ 1) (n=15).
3. 16 had platelet count < 100 cells/μL and decrease by more than 50% from baseline (eSOFA), but platelet decreased to 50-99 cells/μL (coagulation SOFA score 2) from the baseline of 100-149 cells/μL (coagulation SOFA score 1), indicating only 1-point increase in coagulation SOFA score.
4. 14 had their serum bilirubin levels doubled to 2.0 mg/dL or more (eSOFA), but in 10 patients the bilirubin level increased from 1.2-1.9 to 2.0-5.9 mg/dL (delta SOFA score 1), and in the other 4 patients, the bilirubin level increased from 2.0-5.9 to 6.0-11.9 mg/dL (delta SOFA score 1).
5. 13 had doubled creatinine or 50% or more decrease of eGFR (eSOFA), but creatinine reached 1.2-1.9 mg/dL (SOFA score 1).

**Table S2** Frequency of SOFA/eSOFA organ dysfunctions in patients meeting Sepsis-3 or eSOFA criteria

| SOFA/eSOFA organ dysfunctions  n(%) | Sepsis-3 (+)  n=935 | eSOFA (+)  n=573 | Sepsis-3 (+)  eSOFA (-)  n=460 | Sepsis-3 (-)  eSOFA (+)  n=98 |
| --- | --- | --- | --- | --- |
| Cardiovascular | 296(31.7%) | 210(36.6%) | 32(7%) | 0 |
| Respiratory | 675(72.2%) | 233(40.7%) | 302(65.7%) | 24(24.5%) |
| Renal | 380(40.6%) | 114(19.9%) | 150(32.6%) | 13(13.3%) |
| Hepatic | 220(23.5%) | 133(23.2%) | 73(15.9%) | 14(14.3%) |
| Coagulation | 447(47.8%) | 112(19.5%) | 195(42.4%) | 16(16.3%) |
| Neurologic or lactate | 337(36.0%) | 217(37.9%) | 94(20.4%) | 30(30.6%) |

For Sepsis-3(+) and Sepsis(+)/eSOFA(-) patients, each cell represents the number(percentage) of sepsis patients who had 2 or more SOFA points in each organ dysfunction category, with “Neurologic” referring to neurologic SOFA score. For eSOFA(+) and Sepsis(-)/eSOFA(+) patients, each cell represents number(percentage) of sepsis patients who had 1 or more eSOFA points in each eSOFA organ dysfunction category, with “Cardiovascular” referring to vasopressor initiation, “Respiratory” referring to mechanical ventilation initiation, “Renal” referring to doubling in creatinine, “Hepatic” referring to doubling in bilirubin to ≥ 2.0 mg/dL, “Coagulation” referring to decrease in platelets by ≥ 50% to < 100 cells/µL, and “Lactate” referring to lactate ≥ 2.0 mmol/L.

**Figure S1** Frequency of SOFA or eSOFA organ dysfunctions in Sepsis-3+/eSOFA+ sepsis patients

This figure shows the frequency of organ dysfunctions in patients meeting both Sepsis-3 and CDC Adult Sepsis Event eSOFA criteria. The blue bars represent the percentage of sepsis patients who had 2 or more SOFA points in each SOFA organ dysfunction category, with “Neurologic” refers to neurologic SOFA score. The red bars indicate the percentage of sepsis patients who had 1 or more eSOFA points in each category, with “Cardiovascular” referring to vasopressor initiation, “Respiratory” referring to mechanical ventilation initiation, “Renal” referring to doubling in creatinine, “Hepatic” referring to doubling in bilirubin to ≥ 2.0 mg/dL, “Coagulation” referring to decrease in platelets by ≥ 50% to < 100 cells/µL, and “Lactate” referring to lactate ≥ 2.0 mmol/L.

**Table S3** Risk models of in-hospital mortality

|  |  |  | Model 1 | Model 2 | Model 3 | Model 4 |
| --- | --- | --- | --- | --- | --- | --- |
| Variables | Control | Case | Adjusted OR^b^ (95%CI) | Adjusted OR^c^ (95%CI) | Adjusted OR^d^ (95%CI) | Adjusted OR^d^ (95%CI) |
| Age groups (years), n |  |  |  |  |  |  |
| 18-64 | 381 | 25 | 1.00(Reference) | 1.00(Reference) | 1.00(Reference) | 1.00(Reference) |
| 65-84 | 633 | 189 | 4.56(2.95-7.05) | 3.59(2.30-5.63) | 3.36(2.13-5.30) | 2.84(1.73-4.67) |
| ≥85 | 349 | 139 | 6.08(3.88-9.54) | 4.25(2.66-6.78) | 3.84(2.37-6.20) | 2.88 (1.71-4.86) |
| Gender, n |  |  |  |  |  |  |
| Male | 777 | 211 | 1.14(0.89-1.45) | 1.20(0.93-1.54) | 1.20(0.93-1.55) | 1.00(0.75-1.34) |
| BMI (kg/m^2^), n |  |  |  |  |  |  |
| 18.5-24.9 | 395 | 57 |  | 1.00(Reference) | 1.00(Reference) | 1.00(Reference) |
| <18.5 | 87 | 28 |  | 2.21(1.31-3.75) | 2.38(1.39-4.09) | 2.33(1.26-4.32) |
| >24.9 | 227 | 32 |  | 1.13(0.70-1.82) | 1.16(0.72-1.89) | 1.09(0.63-1.88) |
| Bedridden | 654 | 236 |  | 1.89(1.36-2.64) | 1.77(1.26-2.48) | 1.85(1.26-2.70) |
| Charlson Comorbidity Index^a^ |  |  |  | 1.25(1.16-1.33) | 1.25(1.16-1.34) | 1.26(1.16-1.37) |
| Site of infection, n |  |  |  |  |  |  |
| Pneumonia | 784 | 250 |  |  | 3.93(2.13-7.26) | 3.14(1.63-6.06) |
| Urogenital tract infection | 91 | 10 |  |  | 1.30(0.83-2.04) | 1.64(1.01-2.66) |
| Intra-abdominal infection | 146 | 31 |  |  | 1.61(1.26-2.05) | 1.27(0.98-1.65) |
| Skin/soft tissue infection | 27 | 1 |  |  | 0.90(0.53-1.54) | 0.99(0.57-1.73) |
| Septicemia/bacteremia | 26 | 5 |  |  | 1.24(0.98-1.56) | 1.18(0.92-1.51) |
| Two or more infections | 83 | 44 |  |  | 1.39(1.23-1.56) | 1.30(1.14-1.48) |
| Groups, n |  |  |  |  |  |  |
| Sepsis-3-/eSOFA- |  |  |  |  |  | 1.00(Reference) |
| Sepsis-3+/eSOFA- |  |  |  |  |  | 2.09(1.35-3.25) |
| Sespsis-3-/eSOFA+ |  |  |  |  |  | 2.45(1.27-4.73) |
| Sepsis-3+/eSOFA+ |  |  |  |  |  | 17.20(11.52-25.67) |
| AIC |  |  | 1660 | 1602 | 1566 | 1258 |

*Abbreviation*: *BMI* body mass index, *AIC* Akaike information criterion

^a^Odds ratios correspond to a comparison between patients separated by 1-unit change in Charlson Comorbidity Index score.

^b^Adjusted for age categories and gender

^c^Adjusted for age categories, gender, BMI and Charlson Comorbidity Index

^d^Adjusted for age categories, gender, BMI, Charlson Comorbidity Index and site of infection
